# Pseudomyxoma peritonei – two novel orthotopic mouse models portray the PMCA-I histopathologic subtype

**DOI:** 10.1186/1471-2407-7-116

**Published:** 2007-06-30

**Authors:** Kjersti Flatmark, Wenche Reed, Thomas Halvorsen, Olaf Sørensen, Johan N Wiig, Stein G Larsen, Øystein Fodstad, Karl-Erik Giercksky

**Affiliations:** 1 Department of Surgical Oncology, Rikshospitalet-Radiumhospitalet Medical Centre, Montebello, 0310 Oslo, Norway; 2 Department of Tumor Biology, Institute for Cancer Research, Rikshospitalet-Radiumhospitalet Medical Centre, Montebello, 0310 Oslo, Norway; 3 Department of Pathology, Rikshospitalet-Radiumhospitalet Medical Centre, Montebello, 0310 Oslo, Norway; 4 Norwegian Radium Hospital Faculty Division, University of Oslo, 0310 Oslo, Norway

## Abstract

**Background:**

Pseudomyxoma peritonei (PMP) is a rare malignant disease, most commonly originating from appendiceal lesions and characterized by accumulation of mucinous tumor tissue in the peritoneal cavity. Since the disease is infrequent, the task of carrying out studies of treatment efficacy and disease biology in the clinical setting is challenging, warranting the development of relevant *in vitro *and *in vivo *PMP models.

**Methods:**

Human tumor tissue was implanted in the peritoneal cavity of nude mice to establish two orthotopic models exhibiting noninvasive intraperitoneal growth without metastasis development.

**Results:**

Xenograft tissues have retained essential properties of the original human tumors, such as macro- and microscopic growth patterns, mucin production as well as expression of carcinoembryonal antigen, cytokeratins 20 and 7 and the proliferation marker pKi67. Upon microscopic examination, the human tumors were categorized as the PMCA-I (peritoneal mucinous carcinomatosis of intermediate features) subtype, which was conserved through 14 examined passages in mice, for the first time modeling this particular histopathologic category.

**Conclusion:**

In conclusion, two novel orthotopic models of human PMP have been established that consistently portray a distinct histopathologic subtype and reflect essential human tumor properties. Xenografts can easily and reproducibly be transferred to new generations of mice with acceptable passage periods, rendering the models as attractive tools for further studies of PMP biology and treatment.

## Background

Pseudomyxoma peritonei (PMP) is a rare malignant disease characterized by progressive, abundant, multifocal accumulation of mucinous tumor tissue in the peritoneal cavity, essentially without extraperitoneal growth and metastasis development. Patients usually present with abdominal distension due to tumor growth as their main symptom, and untreated, progressive growth over several years will lead to abdominal compression and a fatal outcome. The intraabdominal origin of PMP has been a controversial issue, particularly versus borderline ovarian tumors. Consensus now, however, based on immunohistochemical and molecular studies, is that PMP is of intestinal origin, most commonly originating from appendiceal lesions [1-5].

Cytoreductive surgery, which ideally includes complete removal of all macroscopic lesions, remains the cornerstone of treatment for PMP, and the extent of cytoreduction has been reported to have an impact on disease survival [6,7]. Surgery has during the latter decades been supplemented by intraperitoneal chemotherapy, most commonly in the form of hyperthermic intraperitoneal chemotherapy (HIPEC) using Mitomycin C. HIPEC has been implemented as standard therapy in many treatment centers, although its superiority to surgery alone has not been assessed in randomized controlled trials. The addition of HIPEC increases treatment morbidity and mortality, thus warranting careful patient selection and continuous assessment of potential factors to enable prediction of prognosis based on pretreatment information. Three histopathological subtypes were identified in a series of 109 PMP patients receiving cytoreductive surgery and intraperitoneal chemotherapy: disseminated peritoneal adenomucinosis (DPAM), peritoneal mucinous carcinomatosis (PMCA) and an intermediate histological type (PMCA-I/D) [8]. Patients with DPAM had 5- and 10-year survival rates of 75% and 68%, respectively, whereas corresponding survival rates for PMCA-I/D were 50% and 21%, and for PMCA 14% and 3%. Similar results were recently reported in two different patient populations [9,10], and the importance of histological classification as one of few predictive prognostic variables in PMP was further substantiated by an alternative, dichotomous classification system [11], additionally emphasizing an unresolved controversy in PMP, namely one of nomenclature.

Studies of PMP biology and evaluation of different treatment regimens have been hampered by the lack of appropriate *in vitro *and *in vivo *models, leaving researchers to draw conclusions from clinical studies only, with the obvious limitations this confers in such a rare condition. Animal models of PMP should mimic the peritoneal growth pattern and lack of metastasis development and also reflect histopathological classification. Since standard treatment of PMP is essentially local, involving surgery and intraperitoneal chemotherapy, tumor models should be orthotopic, that is, established as intraperitoneal (i.p.) rather than subcutaneous (s.c.) xenografts. To our knowledge, no models have so far been described to complete these criteria. The present report describes the establishment and characterization of two novel orthotopic xenograft models in mice from human PMP tissue.

## Methods

### Patients

Tumor tissue was acquired from arbitrarily selected tumor areas from patients undergoing surgical treatment for pseudomyxoma peritonei at The Norwegian Radium Hospital, after obtaining written informed consent. The study was approved by the Regional Committee for Medical Research Ethics of Southern Norway. No chemotherapy was given to any of the patients prior to the main surgical procedure.

Patient 1:

The surgical specimen used to establish the PMP-1 model was obtained from an 83-year-old woman whose primary surgery had taken place 6 years previously when an isolated appendiceal mucinous cystadenoma was removed by ileocecal resection. She presented with abdominal distension and a preoperative CT scan indicated multiple, large tumor foci throughout the abdominal cavity, upon which palliative surgical removal of large amounts of abdominal mucinous tumor tissue was performed.

Patient 2:

Tissue for the PMP-2 model was collected from a disseminated abdominal mucinous tumor in a 56-year-old woman. She had been subjected to two previous surgical procedures, the first eight years previously when resection of a mucinous adenocarcinoma of the appendix with a metastatic lesion to the right ovary was performed, and the second two years later when the left ovary with a mucinous cystadenomatous tumor was removed, consistent with a metastatic lesion. The patient experienced abdominal swelling and CT scans indicated a disseminated tumor recurrence. A curative surgical strategy was pursued, including removal of the omentum majus, resection of the transverse colon, splenectomy, cholecystectomy and pelvic peritonectomy.

### Animals

Locally bred female BALB/c nude (nu/nu) mice, 6–8 weeks of age at implantation, were used. The animals were maintained under specific pathogen-free conditions, and food and water were supplied *ad libitum*. Housing and all procedures involving animals were performed according to protocols approved by the animal care and use committee, in compliance with the National Committee for Animal Experiments guidelines on animal welfare. During surgical procedures, mice were anaesthetized using s.c. injections of 0.075 ml/10 g of a mixture of tiletamine, 2.4 mg/ml and zolazepam, 2.4 mg/ml, (Zoletil vet, Virbac Laboratories, Carros, France), xylazine, 3.8 mg/ml, (Narcoxyl vet, Roche, Basel, Switzerland) and butorphanol, 0.1 mg/ml, (Torbugesic, Fort Dodge Laboratories, Fort Dodge, Iowa, USA).

### Tumor models

Fresh tumor tissue was cut into 3 × 3 × 3 mm pieces and implanted either s.c. or i.p. For i.p. implantation a small midline laparotomy was performed and six tumor pieces placed in the peritoneal cavity in both upper and lower abdominal quadrants as well as in both flanks. After implantation, the abdominal wall was closed in two layers with Dexon 5/0. In later i.p. passages, mucinous ascites dominated the growth pattern, and transfer of tissue was in such cases alternatively performed by i.p. injection of 250 μl of mucinous ascites, and no laparotomy was required. Usually, 2–3 mice were implanted or injected per passage.

### Sampling and evaluation

The well being of the mice was carefully monitored and animals were sacrificed when and if signs of disease were detected. In i.p. implanted mice, the main indicator of tumor growth proved to be increased abdominal volume, and if no other symptoms were present they were sacrificed when a distinct increase of abdominal size was detectable, typically 1–3 months after implantation. In s.c. implanted mice, tumors were allowed to reach a maximum of 20 mm in longest diameter provided that the animal had no symptoms from the tumor. Autopsy was performed and macroscopic assessment was made for the presence of tumor tissue or metastatic lesions and sampled for further analyses.

### Histological examination and immunohistochemical staining

Formalin fixed tissue from primary lesions, main surgical specimens as well as samples from all tumor-bearing mice were subjected to haematoxylin-eosin (HE) and alcian blue staining using standard protocols. Histological classification was performed according to Ronnett et al [8]. Additionally, selected samples from each passage through passage 6 were immunostained using monoclonal antibodies against carcinoembryonal antigen (CEA), cytokeratin 7 (CK7), cytokeratin 20 (CK20) and Ki67 (Table 1) using the Dako EnVision™ + detection system according to manual (DAKO, Glostrup, Denmark). For all antibodies, positive controls were included with satisfactory results. The number of immunoreactive tumor cells was semi quantitatively evaluated: 0 (no positive cells), <10%, 10–50%, >50%, 100%. Positive staining for CEA, CK7 and CK20 was noted as cytoplasmic or membranous and for Ki67 nuclear staining was assessed. As these lesions often are paucicellular, immunohistochemistry was performed on sections from the block with the most cellular tumor tissue, and the entire section was then evaluated to establish an immunoreactive score.

**Table 1:** Primary antibodies and immunostaining conditions used for immunohistochemical studies in primary tumors, main surgical specimens and xenografts.

Antibodies	Catalogue	Pretreatment	Dilution	Positive controls
CEA	Dako M7072	Low pH	1/100	Intestinal mucosa Colon cancer
CK20	Progen 61026	Tris-EDTA	1/100	Intestinal mucosa Colon cancer
CK7	Dako M7018	Tris-EDTA	1/200	Intestinal mucosa Colon cancer

Ki67	Dako M7240	Tris-EDTA	1/150	Appendix

## Results

### Tumor growth in animal models

**Figure 1:**
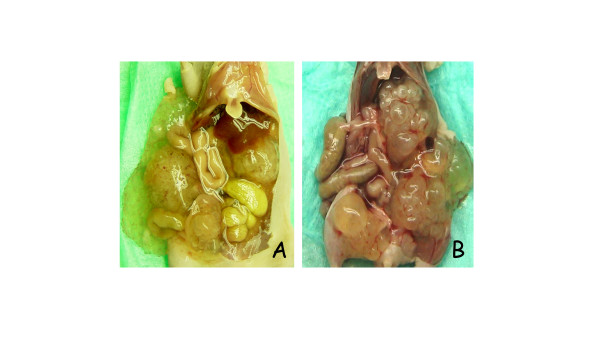
**Autopsy photographs illustrating macroscopic growth patterns in tumor models**. A) In passage 4 of PMP-1 some non-adherent mucinous ascites was observed , but adherent tumor tissue dominated the picture with tumor lesions identified on the surface of the urinary bladder, liver hilum and mesentery of the small intestine. B) Passage 0 of the PMP-2 model exhibited almost exclusively large, "solid" tumors adherent to intraperitoneal surfaces such as liver hilum, retroperitoneum and urinary bladder.

The typical initial i.p. growth pattern was characterized by mucinous ascites accompanied by a variable number of "solid" mucinous tumor lesions of varying size (Figure 1). The solid tumor components were attached to the peritoneum and the serosa of intraperitoneal organs, such as urinary bladder, omentum majus, ovaries, liver- and splenic hilum, but invasive growth was never observed in HE sections. No metastatic lesions were observed in any animal throughout the entire series. After several passages, the growth pattern in both models changed to be increasingly dominated by mucinous ascites with less frequent appearance of lesions adhering to abdominal organs. From passage 6, parallel grafting was performed by injection of mucinous ascites, and this mode of passage resulted in some reduction of the time period between grafting and discernible abdominal distension. The take rate was close to 100% regardless of subculturing strategy, but with the injection approach, tumor growth was more synchronized within each generation. Using the injection approach, we have also succeeded in reestablishing i.p. growth from harvested material that has been stored at -196°C for 3–6 months. We were unable to establish s.c. growth for the PMP-2 model, but PMP-1 did grow when implanted s.c., although more slowly than in the i.p. location. Thus, the s.c. xenografts have presently reached passage 5, whereas both i.p. models are in passage 14. In HE and alcian blue stained sections, tumor tissue harvested from the s.c. location did not differ from the i.p. counterparts, and results from the PMP-1 s.c. model will not be presented in further detail.

### Microscopic assessment

**Figure 2:**
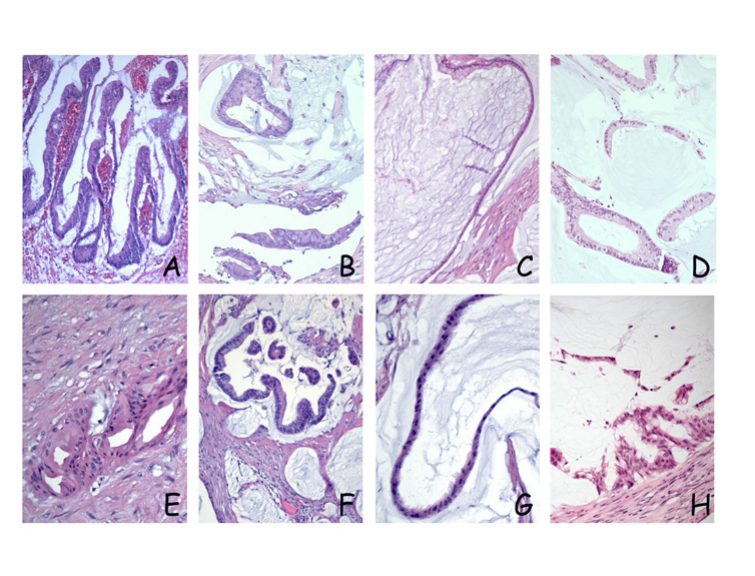
**Microscopic growth assessed by HE staining**. Top panels (A, B, C and D) and bottom panels (E, F, G and H) represent sections from the PMP-1 and PMP-2 models, respectively. In both patients, primary tumor manifestations were appendiceal lesions; (A) Patient 1: cystadenoma of the appendix with low grade atypia. (E) Patient 2: mucinous adenocarcinoma, the selected section illustrating an area of invasive growth in the appendiceal wall. Peritoneal lesions from the main surgical specimens were remarkably similar in the two patients, exhibiting focal areas of cribriform growth and nuclear stratification (B and F), but in both cases, the histopathologic picture was dominated by strips of bland epithelium lining large accumulations of extracellular mucin (C and G). In xenografts from both models a similar histological growth pattern was observed, with adenomucinosis as the dominating manifestation, but with focal areas of nuclear stratification and cribriform growth, leading to classification as PMCA-I (D and H).

**Figure 3:**
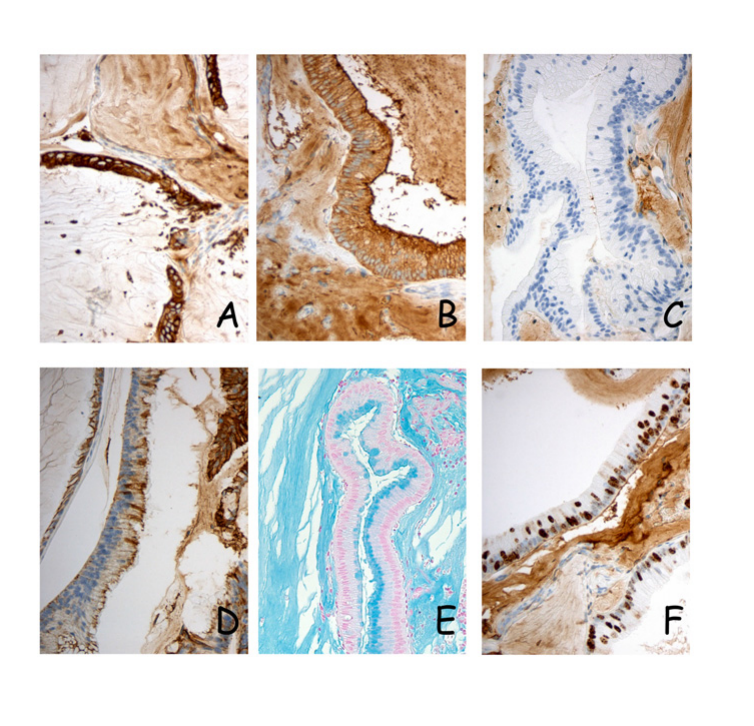
**Immunohistochemical and alcian blue staining of xenograft tissues**. Consistently high membranous and cytoplasmic expression of CEA and CK20 (panels A and B, respectively) was observed in primary tumors, main surgical specimens and in all specimens harvested from the first six animal passages, here illustrated by sections from PMP-1 passage 1. CK7, on the other hand was hardly expressed in the PMP-1 series (panel C), whereas the PMP-2 model (passage 2) exhibited high expression of this cytokeratin (panel D), showing a distinct phenotypic difference between otherwise very similar tumors. Intra- and extracellular mucin was present in all examined sections (panel E). A high fraction of pKi67 positive cells was detected, and in this PMP-1 passage 3 tumor 10–50% of tumor cell nuclei were stained (panel F).

The primary lesion of patient 1 was an appendiceal mucinous cystadenoma with low grade atypia, whereas the patient 2 primary lesion had the characteristics of a mucinous adenocarcinoma. The peritoneal lesions from the two main surgical specimens were, however, remarkably similar upon microscopic examination (Figure 2). The tumors were dominated by extracellular mucin with strips of bland, adenomatous, mucinous epithelium, but focal areas were observed, exhibiting more abundant, glandular epithelium with nuclear stratification, rendering the microscopic picture slightly more "aggressive" than DPAM and consistent with the PMCA-I category. No parenchymal infiltration or signet ring cells were observed, hardly any mitoses were seen and atypia was minimal. The histological picture observed in xenografts from both models was almost identical to the original human tumors, consistently portraying the PMCA-I histological subgroup, although the general impression was that the xenografts were slightly richer in epithelial cells (Figure 2, panels D and H). All examined tumors were shown to contain intra- and extracellular mucin visualized by Alcian blue staining on sections from selected passages (Figure 3E).

### Immunohistochemistry (Table 2 and Figure 3)

**Table 2:** Results of immunohistochemical staining performed on sections from the patients' primary tumors, the surgical specimens implanted in the animals as well as the first passages in the two animal models (p0 – p6).

		**CEA**	**CK20**	**CK7**	**Ki 67**
PMP-1	Primary tumor	100% m/c	100% m/c	0	10–50%
	Main surgical specimen	100% m/c	100% m/c	0	10–50%

	p0	100% m/c	100% m/c	<10% m	10–50%
	p1	100% m/c	100% m/c	0	>50%
	p2	100% m/c	100% m/c	0	10–50%
	p3	100% m/c	100% m/c	0	10–50%
	p4	100% m/c	100% m/c	0	10–50%
	p5	100% m/c	100% m/c	0	10–50%
	p6	100% m/c	100% m/c	<10% m	10–50%

PMP-2	Primary tumor	>50% m/c	100% m/c	10–50% m/c	<10%
	Main surgical specimen	100% m/c	100% m/c	100% m/c	10–50%

	p0	100% m/c	100% m/c	100% m/c	>50%
	p1	100% m/c	100% m/c	100% m	>50%
	p2	100%m/c	100% m/c	100% m	>50%
	p3	100% m/c	100% m/c	100% m/c	>50%
	p4	100% m/c	100% m/c	>50% m	10–50%
	p5	100% m/c	100% m/c	100% m	>50%
	p6	100% m/c	100% m/c	100% m	>50%

Numbers indicate percentage of cells stained in the section.

m = membranous staining c = cytoplasmic staining

A consistently high expression was seen for CEA and CK20 in both tumors as well as in tumor tissue harvested from the animals. The PMP-1 model was essentially negative for CK7 expression, with the exception of slight membranous staining in two of the passages, whereas PMP-2 exhibited a relatively consistent, high expression of this cytokeratin. A Ki67 positive cell fraction of 10–50% in the primary tumor was conserved throughout the animal passages in the PMP-1 animal model. In the PMP-2 model, low and intermediate Ki67 positive cell fractions were found in the primary tumor and main surgical specimen, respectively, whereas in tissue harvested from the animals the positive fraction was consistently high (>50%).

The two intestinal tumor markers, CEA and CK20, were highly expressed in both primary tumors, main surgical specimens and in all specimens harvested from the first six animal passages, with membranous and cytoplasmic staining observed in 100% of tumor cells in all but one section. CK7, a cytokeratin commonly associated with gynecological malignancies, was essentially not detectable in the PMP-1 model, with the exception of slight membranous staining in two of the murine passages (<10% of tumor cells). In contrast, PMP-2 exhibited high expression of this cytokeratin in all examined specimens, with predominantly membranous staining observed in the xenografts, whereas the human equivalents displayed combined cytoplasmic and membranous staining. Nuclear presence of the proliferation marker pKi67 was detected in 10–50% of cells in the PMP-1 primary tumor and main surgical specimen, and this feature was conserved throughout the animal passages. In the PMP-2 model, the pKi67 expression was less consistent, with low and intermediate Ki67 positive cell fractions in the primary tumor and main surgical specimen, respectively, whereas positive fraction in tumor tissue from the animals was consistently high (>50%).

## Discussion

Human tumor tissue from two patients with PMP was successfully implanted into the peritoneal cavity of nude mice to establish two novel, orthotopic animal models. Xenografts have retained fundamental properties of the implanted specimens through several passages, harboring strict peritoneal, noninvasive growth, absence of metastases, and almost identical morphologic appearance of tumor tissue and immunohistochemical staining patterns.

The macroscopic growth pattern of the models closely resembled their human counterparts, as tumor expansion in the i.p. site was characterized by strict peritoneal growth without invasion of peritoneal surfaces and no metastasis development. Also, much like patients afflicted with PMP, the condition was asymptomatic in the mice, to be detected only by increasing abdominal distension. An interesting difference between murine and human tumors was the xenograft tendency to grow as unattached, mucinous ascites, whereas the tissue harvested from the respective patients was rather fibrous in consistency and more firmly adherent to intraabdominal peritoneal surfaces, although without invasive growth. There is no obvious explanation for this observation; however, since microscopic assessment and immunohistochemical analysis revealed almost identical findings in human and xenograft tissues, essential phenotypes of the human tumors seem to have been conserved. For practical purposes, this property has been quite useful, since regrafting by injection of mucinous ascites has eliminated the need for laparotomy, sparing the mice from surgical trauma and being less time consuming. If large series were to be attempted, for instance to test efficacy of chemotherapeutic agents, this might constitute a great advantage.

Histological assessment, comparing tissue architecture, cell morphology and mucin distribution showed convincing similarity between implanted patient tissue and harvested xenografts. Although the primary lesion in the PMP-2 patient was diagnosed as a mucinous adenocarcinoma and the PMP-1 primary lesion was non-invasive, the resulting peritoneal manifestations of both main surgical specimens displayed characteristics of PMCA-I, an intermediary histologic category harboring mainly DPAM features, but with focal areas containing more atypical epithelium [8]. Although tumors from the animals were slightly richer in epithelial cells, the PMCA-I picture was representatively preserved in animal passages, for the first time providing models specifically portraying this histopathological subtype.

A very limited number of studies have addressed phenotypic traits of PMP cells, and most of these have been immunohistochemical studies focused on clarifying the intra-abdominal origin of the disease. In this study, immunohistochemical analyses were undertaken for two main reasons, to compare expression patterns with previously published results and to see whether phenotypic characteristics of implanted tissue would be conserved in the models. CEA is widely used as a tumor marker in colorectal cancer, and previous studies have shown that serum levels are also often elevated in patients with PMP [12]. Hence, the high expression of CEA in tumor cells in human tumors and xenografts is not surprising. The uniformly high expression of CK20 is also in agreement with previously published results and with the appendiceal origin of PMP [1]. Implanted tissue in the PMP-1 model was negative for CK7 expression and only slight (<10%) membranous staining was seen in two of the murine passages, whereas the PMP-2 model exhibited high membranous CK7 expression in all examined sections. While this cytokeratin is more commonly associated with ovarian malignancies, previous immunohistochemical studies have shown that although tumors derived from the appendix in general are negative for CK7; in some cases, appendiceal adenomas and derived peritoneal lesions may be CK7 positive [1,2,13]. Taken together, the expression patterns of CEA, CK20 and CK7 conform well to previous observations in PMP, and findings within each of the two models were consistent throughout the examined passages.

The analysis of pKi67 expression is a well-established method to determine the fraction of actively proliferating cells in a tissue, and the fraction of Ki67 positive cells has been suggested as a marker to predict both disease prognosis and therapy response [14]. Since PMP lesions have not been studied with respect to pKi67 expression, assumptions about the expected frequency of positive cells cannot be made *a priori*. Examined sections from surgical specimens used for implantation exhibited in both cases positive staining for pKi67 in 10–50% of cells, indicating that these were indeed actively proliferating tumors. In the PMP-1 model the fraction of pKi67-expressing cells was almost identical in all murine passages, whereas PMP-2 xenografts exhibited higher scores than the implanted tissue. The fairly high Ki67 score was somewhat surprising considering that almost no mitotic figures were identified in tumor epithelium in HE sections, but consistent with the clinical presentation in the patients as well as with the observed rapid intraperitoneal growth in mice.

Yakushiji et al were able to grow tissue from an ovarian tumor both i.p. and s.c. in nude mice, and based on microscopic appearance it was classified as "ovarian pseudomyxoma peritonei" [15]. Although no detailed histopathological description was provided, published images certainly suggest the presence of mucinous tumor tissue, and the described growth patterns, dependable take rates and favored i.p. growth seem well in keeping with our findings. Similar to our findings, mucinous deposits were reported to be positive for alcian blue staining, and serum levels of CEA were elevated in tumor bearing mice compared to control mice, indicating relevance of CEA in this model as well. Interestingly, the authors also succeeded in utilizing the model to assess the effect of i.p. injections of cisplatin and adriamycin on tumor growth, thus confirming the usefulness of this strategy for in vivo studies of i.p. chemotherapeutic efficacy.

## Conclusion

Two novel orthotopic models of human PMP were established in mice, in which macroscopic growth pattern, the specific histopathologic subtype and immunohistochemical expression pattern of the implanted material were well preserved. Xenograft material can easily and reproducibly be transferred to new generations of mice with acceptable passage periods, rendering the models as attractive tools for further studies of PMP biology and treatment.

## Competing interests

The author(s) declare that they have no competing interests.

## Authors' contributions

KF conceived of the study, participated in its design, participated in the animal experiments and drafted the manuscript. WR carried out the histopathological studies and participated in drafting the manuscript. TH carried out the animal studies. OS, JNW, SGL, ØF and KEG participated in the design of the study and helped draft the manuscript. All authors read and approved the final manuscript.

## Pre-publication history

The pre-publication history for this paper can be accessed here:


